# Editorial: Cytokine and cytokine receptor-based immunotherapies: Updates, controversies, challenges, and future perspectives

**DOI:** 10.3389/fimmu.2022.985326

**Published:** 2022-07-26

**Authors:** Jun Wang, Brent Johnston, Pedro Berraondo

**Affiliations:** ^1^ Department of Microbiology and Immunology, Dalhousie University, Halifax, NS, Canada; ^2^ Department of Pediatrics, Dalhousie University, Halifax, NS, Canada; ^3^ Beatrice Hunter Cancer Research Institute, Halifax, NS, Canada; ^4^ Canadian Center for Vaccinology, Halifax, NS, Canada; ^5^ Program of Immunology and Immunotherapy, Cima Universidad de Navarra, Pamplona, Spain; ^6^ Navarra Institute for Health Research (IDISNA), Pamplona, Spain; ^7^ Centro de Investigación Biomédica en Red de Cáncer (CIBERONC), Madrid, Spain

**Keywords:** interleukin-13 receptor subunit alpha-2, cardiotrophin-like cytokine factor 1, transforming growth factor beta, tumor necrosis factor, C-C chemokine receptor type 2, chemokine (C-X-C motif) ligand 13, interleukin 34, IFN-α

Cytokines are critical in mediating intercellular communications and shaping the tumor microenvironment. Scientific advances made in the last few decades have revealed pivotal roles for specific cytokines in regulating innate and adaptive immune responses against cancer, with some molecules demonstrating clear anti-tumor or pro-tumor effects and others displaying pleiotropic effects (1, 2). In this collection of five review articles and two research articles, 41 authors have contributed to this Research Topic, discussing the challenges, controversies and future perspectives for cytokine and cytokine receptor-based immunotherapies.

A number of immune-stimulatory cytokines, including IFN-α, GM-CSF, IL-2, IL-7, IL-12, IL-15, IL-18 and IL-21, have been explored as anti-tumor agents, among which IFN-α and IL-2 are approved by FDA for treating various malignancies (3, 4). While many of these anti-tumor cytokines achieved limited success as single therapeutic agents in clinical trials, their immune-stimulatory properties have engaged strong interest in using these cytokines in combination therapies with checkpoint inhibitors and other therapies. Atallah-Yunes and Robertson review the biology and recent advances of several immune-stimulatory cytokines, with a specific focus on studies in lymphoma.

Conversely, T helper 2 cytokines like IL-4 and IL-13 are pro-tumor cytokines and their cognate receptors have been recognized as novel therapeutic targets (5). Due to the overexpression of interleukin-13 receptor subunit alpha-2 (IL-13Rα2) on a variety of tumors and its association with tumor progression, this receptor has received interest as a target of cancer immunotherapy. Knudson et al. review therapeutic approaches to targeting IL-13Rα2. Receptor targeting peptides, ligands engineered for selectivity, and antibodies coupled to cytotoxic molecules have generated promising results in pre-clinical models and some clinical trials. CAR-T cells targeting IL-13Rα2 have generated mixed results due to poor persistence and selection for IL-13Rα2-negative tumor recurrence. IL-13Rα2 has been targeted as one of several antigens in multi-target cancer vaccines and IL-13Rα2 therapeutics are likely to work better in combination therapies incorporating other modalities.

The IL-6 family cytokine, cardiotrophin-like cytokine factor 1 (CLCF1), acts on the tumor microenvironment to promote tumor cell stemness and a pro-tumorigenic microenvironment (6, 7). Jiang et al. used bioinformatic analysis to show increased CLCF1 expression in glioma patients, a feature that correlated with poor survival outcome in two cohorts and had greater prognostic use than IL-6. CLCF1 correlated positively with higher regulatory T cell (Treg) levels and higher expression of immune checkpoint markers (PD1/PD-L1). However, patients with low CLCF1 were more likely to respond to anti-PD-1 therapy. This suggests that additional factors act in the microenvironment to regulate immunity.

Notably, many pleiotropic cytokines display complicated or opposing roles in cancer, with many exhibiting anti-tumor and pro-tumor activities in a stage-dependent manner. This may be attributed to dynamic changes in intratumoral immune cells (8) and intricate positive and negative feedback signaling networks within the tumor microenvironment (9, 10). Liu et al. review the literature surrounding the respective roles of transforming growth factor beta (TGF-β) and tumor necrosis factor (TNF-α) in tumor formation and the combinational effects of the two cytokines in various aspects of tumorigenesis, including cell proliferation, apoptosis, inflammation, extracellular matrix and genomic instability. While TGF-β and TNF-α display synergistic effects in certain settings and certain cell types, they antagonize each other in other conditions and different cell types. A yin-yang theory is proposed to describe the crosstalk effect between TGF-β and TNF-α, in which they restrict and balance each other.

Chemokines and chemokine receptors play critical roles in leukocyte trafficking and the composition of tumor-infiltrating lymphocytes, as such, chemokine/chemokine receptors have been explored as anti-cancer therapeutic targets (11, 12). The C-C chemokine receptor type 2 (CCR2) is expressed by multiple cell types, including monocytes, immature dendritic cells, Tregs, endothelial cells and cancer cells (13). CCR2 and its ligands mediate recruitment and polarization of suppressive myeloid cells (14, 15), recruitment and activity of Tregs (16, 17), and survival and invasion of tumor cells (13). Fei et al. review the role of CCR2 in these processes and outline strategies to target CCR2 for cancer immunotherapy. Targeting CCR2 is likely complicated by redundancies in chemokine and chemokine receptor networks. However, some studies suggest CCR2 antagonism may work better in combination with other therapies including chemotherapeutics, radiation, and checkpoint blockade. The chemokine (C-X-C motif) ligand 13 (CXCL13) and its receptor CXCR5 are among the key chemotactic factors regulating tumor-specific microenvironment in a context-dependent manner (18). Zhang et al. conducted a comprehensive bioinformatic analysis of 33 human cancer datasets from The Cancer Genome Atlas (TCGA) to examine the correlation between CXCL13 expression and clinicopathological characteristics, prognosis, mismatch repair genes (MMRs), microsatellite instability (MSI), tumor mutation burden (TMB), immune cells infiltration, immune-related genes, and the role in tumor immunotherapy. The expression of CXCL13, validated in a local cohort, exhibited a dichotomy in modulating clinical outcomes, with a positive correlation in some cancer types and a negative correlation in others. The expression of CXCL13 also seems to be a good biomarker to predict treatment responses to the checkpoint blockade.

Growth factors are a subtype of cytokines that control cell proliferation and/or differentiation. Colony-stimulating factor 1 (CSF-1) and its receptor (CSF-1R) are well-known for their roles in bone marrow homeostasis, particularly for the development, differentiation and activation of myeloid cells (19). IL-34 is the second ligand for CSF-1R (20), which displays comparable biological activity in macrophage differentiation, but stronger impact on macrophage polarization as compared to CSF-1 (21). Monteleone et al. review the role of IL-34/CSF-1R axis in the pathogenesis of colon cancer. They outline the role of IL-34 in controlling the growth of colorectal carcinoma and differentiation of tumor-associated macrophages. They also discuss the role of IL-34/CSF-1R in promoting cancer resistance to chemotherapy and immunotherapy.

Undoubtedly, cytokine and cytokine-receptor-based immunotherapies hold promise for cancer treatment. In addition to the discovery of new therapeutic targets, our Research Topic highlights a future perspective of this research field in the development and optimization of combination therapies with checkpoint inhibitors and other novel therapies.

## Author contributions

All authors listed have made a substantial, direct, and intellectual contribution to the work and approved it for publication.

## Conflict of Interest

The authors declare that the research was conducted in the absence of any commercial or financial relationships that could be construed as a potential conflict of interest.

## Publisher’s note

All claims expressed in this article are solely those of the authors and do not necessarily represent those of their affiliated organizations, or those of the publisher, the editors and the reviewers. Any product that may be evaluated in this article, or claim that may be made by its manufacturer, is not guaranteed or endorsed by the publisher.
